# Safety and Efficacy of Autologous Bone-Marrow-Derived Mesenchymal Stem Cells for Treating Partial Rotator Cuff Tears: Observational Study with 4-Year Follow-Up

**DOI:** 10.3390/biomedicines14020382

**Published:** 2026-02-06

**Authors:** Nicholas Hooper, Ahmad Alhusen, Hinnah Siddiqui, John Pitts, Prathap Jayaram, Chirstopher J. Williams

**Affiliations:** 1 Department of Physical Medicine and Rehabilitation, Emory University School of Medicine, Atlanta, GA 30307, USA; 2 Loop Medical Center, Chicago, IL 60616, USA; 3 Department of Public Health, University of Pittsburgh, Pittsburgh, PA 15261, USA; 4 Centeno-Schultz Clinic, Broomfield, CO 80021, USA; 5 Department of Orthopedics and Physical Medicine and Rehabilitation, Emory University, Atlanta, GA 30322, USA; 6 Regenexx Cayman, George Town, Grand Cayman KY1-1208, Cayman Islands; 7 Interventional Orthopedics of Atlanta, Atlanta, GA 30329, USA

**Keywords:** regenerative medicine, rotator cuff tears, bone-marrow-derived stem cells, mesenchymal stem cells

## Abstract

**Background/Objectives**: Rotator cuff tears (RCTs) are a leading cause of shoulder pain and disability. Management typically involves conservative measures, such as physical therapy and anti-inflammatory medications, or surgery for full-thickness or refractory tears. Regenerative medicine therapies, including platelet-rich plasma (PRP), platelet lysate (PL), and mesenchymal stem cells (MSCs), show promise as alternative treatment strategies, although long-term outcomes remain under investigation. **Methods**: This cohort included 30 patients with partial rotator cuff tears and were treated with culture-expanded MSC injections. There was no control group. Inclusion criteria included an imaging-confirmed diagnosis of partial-thickness rotator cuff tears. Outcomes were assessed at multiple time points up to 6 years. Pain and function were assessed using the Disabilities of the Arm, Shoulder, and Hand (DASH), a Numeric Rating Scale (NRS), and the modified Single Assessment Numeric Evaluation (SANE)**. Results**: Thirty patients (37 shoulders) were included in the analysis. Significant improvements in the NRS and DASH scores were observed at 3, 6, 12, 18, and 24 months (*p* < 0.01). Twenty-four months post-treatment, the mean NRS and DASH decreased by 2.25 and 15.93 points, respectively, and SANE improved by 60%. At six years, among seven respondents, the mean SANE improvement was 75.54%. During this study, no significant adverse events were reported. **Conclusions**: This study provides the longest known follow-up of MSC therapy for partial-thickness RCTs, finding sustained pain and functional improvements. The findings support further research into MSC-based and combination regenerative therapies as a viable alternative treatment option for partial-thickness rotator cuff tears.

## 1. Introduction

Rotator cuff tears (RCTs) are one of the leading causes of shoulder pain, and they represent a significant proportion of musculoskeletal disability worldwide [1]. Overall, the point prevalence and lifetime estimates of shoulder pain can range from 14 to 67% each year, depending on the setting (i.e., primary care vs. emergency department) with an increasing prevalence observed in middle-aged and older adults [2,3,4,5,6,7]. Rotator cuff disorders are identified as the etiology of pain in up to 70% of patients presenting with shoulder pain [8]. Rotator cuff pathology is the most common upper extremity injury encountered by primary care physicians, physiatrists, and orthopedic specialists in the outpatient setting and in the United States accounts for over 4.5 million office visits and USD 3 billion in healthcare costs annually [9,10] The prevalence of asymptomatic RCTs is high as well, with studies demonstrating an observable prevalence of 20% to 50%, with prevalence increasing with age [11,12]. With nearly a third of asymptomatic RCTs becoming symptomatic over the years, undiagnosed injuries can lead to a significant portion of the population with long-term upper extremity disability.

The accurate diagnosis of RCTs can be accomplished by either point-of-care ultrasonography or magnetic resonance imaging. Comparative imaging studies have demonstrated no significant differences in the diagnostic accuracy between the modalities for partial or full-thickness RCTs [13]. Once properly diagnosed, rotator cuff tears can be further classified as partial-thickness to full-thickness tears with or without retraction of the tendon at the bony attachment site. The current management options for rotator cuff tears vary based on the goals and age of the individual and whether there are significant degenerative changes observed in the glenohumeral joint as well. The primary objectives of treatment are focused on alleviating pain, restoring shoulder function, and preventing tear progression. While conservative measures such as physical therapy, anti-inflammatory medications, and corticosteroid injections may be appropriate options for select patients, they are often be limited by patients’ functional and athletic goals, persistent symptoms, and progression of disease, which may ultimately require surgical intervention [14].

The surgical repair of rotator cuff tears, particularly partial-thickness lesions, has evolved over the past decades with the advent of arthroscopic techniques, suture anchors, and improved rehabilitation protocols [15,16]. However, despite these advances, clinical outcomes remain unpredictable, and the rate of structural failure or incomplete healing is notably high, especially in cases involving poor tendon quality, larger tears, or older patients [17,18,19,20]. Re-tear rates following an uncomplicated surgical repair have been reported in the literature as between 13% and 94% [19]. Therefore, given the large variability in the long-term efficacy of conservative and surgical management options, there is an existing need for effective adjunctive and disease modifying therapies for RCTs.

In recent years, there has been growing interest in the use of regenerative therapies to improve tendon healing and recovery in people with rotator cuff injuries. Mesenchymal stem cells (MSCs) are multipotent stromal cells derived from tissues such as bones, adipose tissue, or tendons. MSCs have the capacity to differentiate into various cell types of the mesenchymal lineage, modulate inflammation, and secrete growth factors that support tissue repair [21]. Hernigou et al. reported that there was a reduced content of MSCs at the bone–tendon interface of the RTC in symptomatic RTC tears versus controls, providing a biological explanation as to why MSC therapy could be important in these injuries [22]. Preclinical studies have demonstrated the ability of MSCs to improve tendon and ligament healing, form neofibrocartilage, and improve bone quality [23]. Early clinical research has supported these findings, with several studies reporting improved healing rates, reduced re-tear risk, and better functional outcomes of rotator cuff repair when MSCs are used as an adjunct [24,25]. Hernigou et al. compared RTC repair with adjunctive bone marrow concentrate (BMC) containing MSCs vs. repair alone in 90 patients, 45 in each group. The RTC plus BMC group demonstrated healing on ultrasound in 100% of the patients at 6 months compared to 67% of those receiving RTC repair alone, and, at 10 years, survival rates were 87% vs. 44% [24]. Gomes et al. reported 14 patients treated with autologous bone marrow MSCs at tendon borders during mini-open repair, with MRI showing preserved tendon integrity in 100% of the cases at 12 months, and functional scores stabilized over 24 months [26,27]. Furthermore, studies suggested a dose–response relationship: higher transplanted MSC counts corresponded with better structural outcomes [24].

Centeno et al. reported a randomized controlled trial of symptomatic RTC tears treated with autologous BMC vs. controlled exercise with outcomes up to 2 years [28]. The BMC group outperformed the exercise-only group at 3 months in patient-reported pain and functional scores, and, 2 years post-treatment, 90% of the BMC-treated patients met the MCID for all outcomes, with 73% showing healing on MRI. Despite these promising findings, there is limited long-term data on clinical outcomes following MSC injection for partial rotator cuff tears, especially regarding pain and functional outcomes, and the need for subsequent surgical intervention. The aim of this study was to retrospectively examine the effectiveness of the ultrasound-guided injection of autologous-culture-expanded MSCs for partial-thickness rotator cuff tears. We evaluated functional and pain outcomes at 3 to 4 years post-procedure and assessed the incidence of subsequent surgical interventions at the most recent patient follow-up.

## 2. Materials and Methods

### 2.1. Study Design

The present study was conducted at a single outpatient-based private practice center. A clinical registry was used to prospectively collect patient outcome data. An informed consent form was signed by all participants prior to participation, and the registry data protocol was approved by the International Cellular Medicine Society (Approval #: ICMS-2014-IR-013, Protocol #: HHG2014-01) as well as registered with Clinicaltrials.gov (NCT03011398). Patients received electronic surveys via ClinCapture (Clinovo Clinical Data Solutions, Sunnyvale, CA, USA) to self-report outcomes and adverse events prior to treatment and at 1, 3, 6, 12, 18, and 24 months, and annually thereafter for up to a period of 20 years post-treatment. Patients were considered lost to follow-up if there was no response after 5 attempts were made electronically. Identified adverse events were reviewed, indexed, and further characterized by the treating physician.

### 2.2. Inclusion and Exclusion Criteria

Inclusion criteria included (1) patients undergoing treatment with autologous-culture-expanded bone-marrow-derived MSCs (aBD-MSC); (2) either MRI or diagnostic Ultrasound imaging confirming a PRCT with or without tendinosis or bursitis; (3) patients enrolled in the registry with completed patient reported outcome measures; (4) treatment completed between January 2018 and January 2020. Search parameters for patient identification within the registry included shoulder pain, rotator cuff tears, labral tears.

Patients were excluded if they (1) were diagnosed with severe glenohumeral osteoarthritis (KL grade 4); (2) had any previous shoulder surgery on the shoulder treated with aBD-MSCs; or (3) had a complete full-thickness rotator cuff tear. All biologic injectates utilized and any additional structures injected at the time of treatment were identified (see Table 1).

### 2.3. Preparation of Biologic Injectates

A detailed description of the bone marrow aspiration, isolation, and expansion technique has been previously published [27,29,30,31].

#### 2.3.1. Autologous-Culture-Expanded-Bone-Marrow Derived MSCs (aBM-MSC)

In brief, a total of 90–120 mL of bone marrow was aspirated from the posterior superior iliac spine under ultrasound or fluoroscopic guidance using an 11-gauge trocar under sterile conditions by an experienced physician. The bone marrow aspirate was collected into heparinized syringes containing 1000 units of heparin (Aurobinda pharma limited-NDC 63739-964-25) per milliliter of the syringe for the prevention of clot formation. Similar to the previously published methods, the aspirated bone marrow was further centrifuged for separation of the buffy coat and seeding of the nucleated cells. MSC colonies were isolated and expanded using animal-origin-free media and 10% autologous PL. After the completion of 2–5 passages, the final cellular product was suspended in autologous platelet lysate in preparation for the treatment. Prepared cells were tested for endotoxins, cellular viability, and cell counts prior to being utilized. Quality control measures required negative endotoxin screening and cellular viability > 90% for all patient samples.

#### 2.3.2. Platelet-Rich Plasma (PRP) and Platelet Lysate (PL)

PRP was prepared from a venous blood draw completed within 24 h prior to the planned treatment, with a blood volume of 60–500 mL, depending on the treatment plan. A two-spin centrifugation method at 300× *g* and 1000× *g* was completed for producing a final product low in red and white blood cells. Additionally, the final product containing the highly concentrated platelets was suspended in platelet-poor plasma (PPP).

On the day of the BMA, approximately 500 mL of venous blood was collected into a blood bag containing 80 mL of anticoagulant citrate dextrose solution A (ACD-A), and the PL to be used for culture expansion was prepared from PRP produced via overnight freezing at −20 °C, which was then thawed and recentrifuged at 1000× *g* with the produced supernatant collected as the final PL product. The PL used for injection was similarly prepared on the day of treatment without overnight freezing using an −80 °C freezer for 5–10 min, followed by a similar thaw and re-centrifugation process as previously mentioned.

### 2.4. Injection Technique

Under sterile conditions, all patients received percutaneous injections under ultrasound guidance using a high-frequency linear transducer (Sonosite Edge, Fujifilm Sonosite, Inc., Bothel, WA, USA) for injection into one or more of the rotator cuff tendons, depending on pre-identified pathology based upon diagnostic imaging results. Injections were conducted with or without fluoroscopic guidance for any additional structures that were treated (i.e., glenohumeral joint) at the same time the RTC tear was injected. The injected aBD-MSCs were a carrier of PL for all patients, with some patients also having additional PRP injected into the targeted structures, depending on the preference of the injecting provider. The structures injected are listed in Table 1.

### 2.5. Outcome Measures

The primary endpoints were the Numeric Rating Score (NRS) and Disabilities of the Arm, Shoulder and Hand (DASH) scores at baseline and each follow-up time point. The modified Single Assessment Numeric Evaluation (SANE) was completed at each follow-up time point. The NRS is a scale for the assessment of pain and ranges from 0 to 10, with 0 indicating no pain and 10 indicating the worst pain possible. The DASH scale is a measure of self-rated upper-extremity disability and symptoms. It is a 30-question inventory that is scored between 0 and 100, with a higher score representing more disability. The modified SANE is used to assess patients’ perceived improvement from the treatment modality in comparison to how they felt prior to treatment on a rating scale from −100 (worsened) through +100 (improved). To align the responses to the standard SANE, negative ratings were set to 0.

### 2.6. Statistical Analysis

The standard deviation (SD) and the mean were used to describe continuous variables. Within-subject changes in the Numeric Rating Scale (NRS) and Disabilities of the Arm, Shoulder and Hand (DASH) scores were evaluated using paired Wilcoxon signed-rank tests comparing baseline to each post-treatment time point. Modified Single Assessment Numeric Evaluation (SANE) scores were analyzed using paired Wilcoxon signed-rank tests comparing the 1-month response to subsequent follow-up time points. To account for multiple longitudinal comparisons within each outcome measure, the Bonferroni correction was applied separately for NRS, DASH, and SANE analyses. Effect sizes were calculated using paired Cohen’s d, with values of 0.20–0.49 considered small, 0.50–0.79 medium, and ≥0.80 large. All tests were two-sided, and adjusted *p*-values < 0.05 were considered statistically significant. All statistics were calculated using R Studio (version 2025.05.01) [32].

## 3. Results

A total of 83 patients were treated for shoulder pathology from 1 January 2018–1 January 2020, and 30 patients met our inclusion and exclusion criteria [see Table 1]. Of those patients, a total of 37 shoulders were treated. Of those included, 73.33% (*n* = 22) were male, while 26.67% (*n* = 8) were female. All treated patients were injected with MSC into one or more of the rotator cuff tendons with PL and possibly PRP as an adjunctive treatment (see Table 1). On average, 4.32 million MSCs were injected into one or more of the varying rotator cuff tendons. In total, a mean of 8.53 million MSCs were injected into various areas of each patient’s shoulder, including intratendinous, intra-articular, or into the labrum (Table 2). Of the rotator cuff tendons, the supraspinatus was injected most frequently (*n* = 35), followed by the subscapularis (*n* = 18), infraspinatus (*n* = 15), and teres minor (*n* = 3). Outside of the rotator cuff tendons, the most frequently injected site was the glenohumeral joint (*n* = 13), followed closely by the labrum (*n* = 10) and AC joint (*n* = 8) (Table 2).

**Figure 1:**
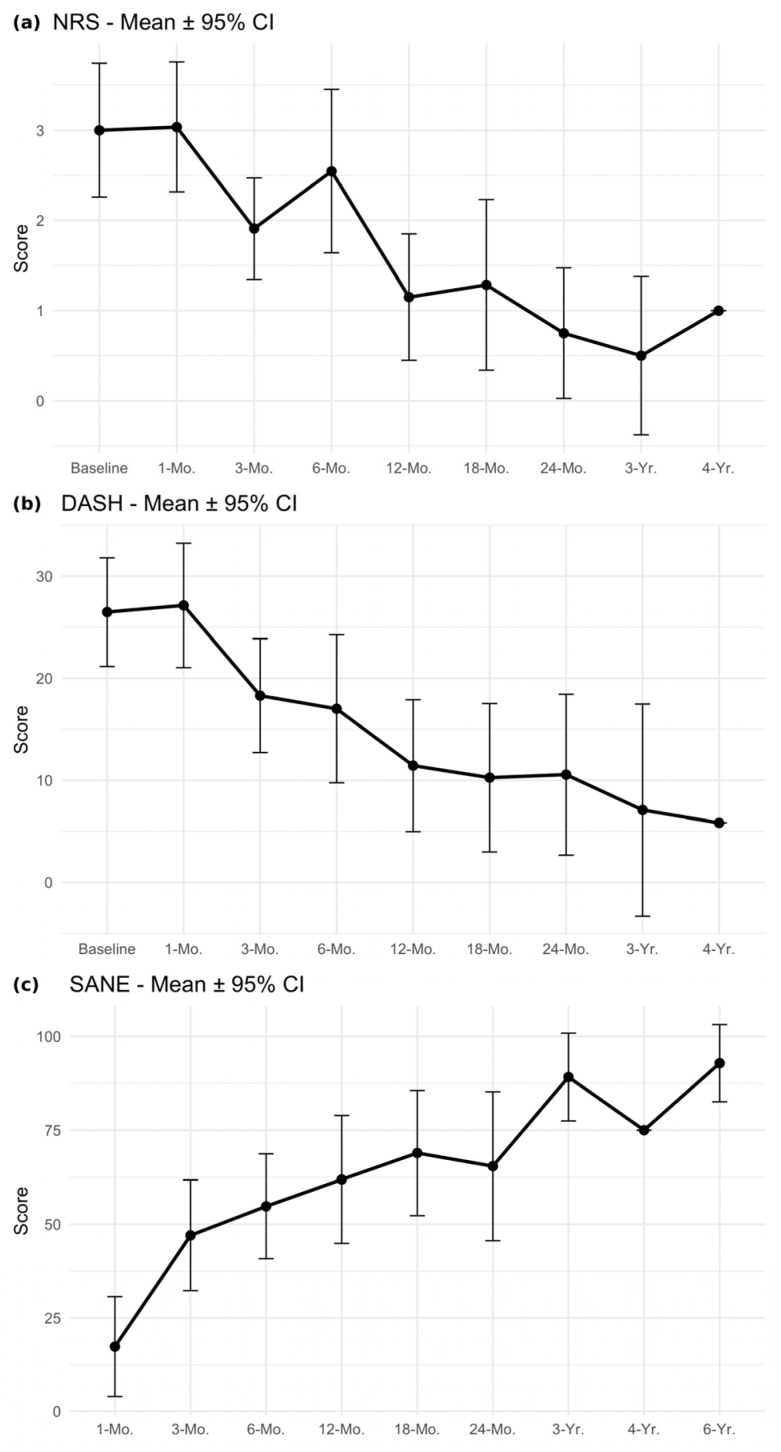
(**a**) Longitudinal trend in NRS means at baseline, 1 month, 3 months, 6 months, 12 months, 18 months, 2 years, 3 years, and 4 years. Bars represent 95% confidence intervals. (**b**) Longitudinal trend in DASH means at baseline, 1 month, 3 months, 6 months, 12 months, 18 months, 2 years, 3 years, and 4 years. Bars represent 95% confidence intervals. (**c**) Longitudinal trend in SANE means at 1 month, 3 months, 6 months, 12 months, 18 months, 2 years, 3 years, 4 years, and 6 years. Bars represent 95% confidence intervals. Abbreviations: NRS; Numeric Rating Score, DASH; Disabilities of the Arm, Shoulder, and Hand, SANE; Single Assessment Numeric Evaluation.

A complete outcome dataset including NRS, DASH, and SANE scores is reported in Table 3 and Table 4. Significant improvement in NRS scores was seen at 3 months, 6 months, 12 months, 18 months, and 24 months when compared to baseline. At 2 years, there was an overall 2.25-point decrease in NRS scores when compared to baseline (*p* ≤ 0.01), which exceeded the minimally clinically important difference (MCID) of two (Figure 1). The improvement in the mean DASH scores reached statistical significance at 3 months, 6 months, 12 months, 18 months, 24 months, and 3 years. At 2 years, there was an overall 20.48-point drop in the average DASH scores when compared to baseline (*p* ≤ 0.01), which exceeded the MCID of 10.83. Lastly, the modified SANE scores significantly improved at 3 months, 6 months, 12 months, 18 months, 24 months, 3 years, and 6 years when compared to the 1-month scores. At 2 years, there was an overall improvement of 45.41 points compared to 1-month scores (*p* ≤ 0.01), which exceeded the MCID of 27.25.

## 4. Discussion

The present study is the first published dataset reporting the long-term outcomes beyond 3 years for the intratendinous treatment of partial-thickness rotator cuff tears (PRCTs) with autologous-culture-expanded mesenchymal stem cells. This retrospective cohort study supports the use of multifactorial regenerative medicine injections for the effective treatment of PRCTs. Of the 30 patients and 37 shoulders treated, all underwent US-guided treatment with culture-expanded MSCs, along with platelet lysate (PL) and/or platelet rich plasma (PRP), of one or more of the rotator cuff tendons. Significant improvements were observed in both pain scores and function outcomes at all time points between 3 months and 2 years. Notably, at 2 years, our cohort revealed an overall improvement of 2.25 points on the NRS, 20.48 points on the DASH, and 45.41 points on the modified SANE, all of which exceeded the MCIDs for these outcomes. All of the patients reporting 1- or 2-year outcomes demonstrated long-term symptom improvement at those time points. Of the patients included, seven provided SANE outcomes at 6 years post-intervention. Of those reporting 6-year data, the average SANE was 92.86, which was an increase of 75.54 points from the 1-month values, demonstrating long-term efficacy.

As previously mentioned, the treatment of rotator cuff tears typically ranges from conservative treatment, such as physical therapy, non-steroidal anti-inflammatory drugs, and activity modification, to various injections and surgery. Despite this, many individuals do not improve and continue to have progression of rotator cuff disease. Several studies have shown that the progression of partial-thickness tears is common, with studies reporting progression of tears in 34–53% of cases [33,34]. Progression of disease, in the absence of treatment, is thought to be due to the extremely low natural healing potential of tendons due to hypocellularity and hypovascularity [35,36,37]. Experimental evidence has shown that the potential therapeutic mechanisms of MSCs lie in their ability to reduce inflammation, enhance tissue remodeling, promote angiogenesis, and improve the mechanical properties of repaired tendons. In in vitro studies, researchers were able to culture BD-MSCs in fibrin gels and spontaneously generate collagen fibrils similar to embryonic tendons [38,39]. Furthermore, a study by Costa-Almeida and colleagues found that MSCs promoted extracellular matrix remodeling and led to the accelerated deposition of type 1 collagen and an increased ratio of type 1 to type 3 collagen [40]. Thus, experimental studies have provided compelling evidence supporting the therapeutic potential of mesenchymal stem cells for partial-thickness rotator cuff tears, which has prompted some clinical human research.

Overall, there are a limited number of trials and RCTs examining the effect of MSCs on rotator cuff tears. A recent review by Hooper et al. found, of 18 studies examining the use of culture- and non-culture-expanded MSCs for the treatment of partial rotator cuff tears, only 5 were RCTs [21]. Additionally, only one of the studies utilized culture-expanded MSCs without surgical intervention for the treatment of partial rotator cuff tears. The study in question by Jo et al. looked at a dose escalation protocol (i.e., 10, 50, and 100 million MSCs) for the utilization of autologous-adipose-derived MSCs to treat partial rotator cuff tears with MRI follow-up up to 2 years [36]. The authors noted significant improvement in the pain and functional outcomes at 2 years and 100% resolution of bursal sided tears on MRI at 2 years. Of the case series, case reports, and cohort studies included in the review, 10 of the 12 found improvements in pain and/or functional outcome scores. A randomized control trial examining adipose-derived MSCs in partial-thickness rotator cuff tears found statistically significant improvements in both pain and function at 12 months when compared to steroids [41]. Similarly, Centeno et al. found that the combination of bone marrow aspirate concentrate and PRP injection also led to significant improvements in pain and function at 12 months compared to exercise therapy [42]. However, some studies have found that MSC injections are not superior to placebo controls. A recent study comparing adipose-derived MSCs plus fibrin glue, fibrin glue only, and saline only for the treatment of partial supraspinatus tears found no significant differences between groups [43].

The present cohort received a mean of 4.32 million culture-expanded aBM-MSCs per targeted rotator cuff tendon (8.53 million per shoulder, including other shoulder structures), which situates our dosing between the relatively low progenitor loads reported with intraoperative BMC interface augmentation and the higher ranges used in culture-expanded adipose protocols. During arthroscopic augmentation, Hernigou et al. observed a dose–response signal in which higher transplanted MSC/progenitor counts at the tendon–bone interface correlated with markedly lower re-tear rates up to 10 years, suggesting that inadequate cell dose is one contributor to structural failure in larger or more chronic tears [24]. Conversely, in non-operative intratendinous approaches, Jo et al. used a dose-escalation design (10, 50, 100 million autologous-adipose-derived MSCs) with the two higher-dose groups reporting more significant clinical improvement and high rates of MRI lesion resolution at 2 years—doses one to two orders of magnitude above our mean per-shoulder delivery [36]. Not all high-dose approaches translate to superior outcomes however an allogeneic-adipose-derived MSC product embedded in fibrin glue did not outperform controls in a randomized trial, underscoring that “effective dose” is not independent of cell provenance (autologous vs. allogeneic), tissue source (bone marrow vs. adipose), viability/potency (e.g., CFU-F frequency), carrier/scaffold, or delivery target (tendon substance vs. tendon–bone interface) [43]. Our findings—durable, clinically meaningful improvements with mid-range doses of autologous-culture-expanded aBM-MSCs delivered intratendinously with PL±PRP—support the emerging consensus that using more cells may be better up to a plateau and that the optimal dose likely differs with cell source and route. Future trials should (i) normalize dose-to-lesion/tendon volume or tear length, (ii) report functional units such as MSC per milliliter of injectate, and (iii) directly compare pragmatic aBM-MSC doses in the 5–10 million range to higher adipose-derived doses (50–100 million) to determine whether higher cell numbers, different tissue sources, or autologous versus allogeneic origin primarily drive structural and clinical benefits in partial-thickness tears [24,36,42,43].

Studies have examined the efficacy of both MSCs and PRP as adjuncts to arthroscopic repair. A recent review by Carola et al. (2025) found that there were seven level 1 to level 4 studies examining the use of BMAC as augmentation for rotator cuff repair [44]. Of those studies, four out of seven reported statistically significant improvements in at least four different outcome scores. However, other studies have found no significant differences in pain and function between BMAC groups and controls [24,45]. In addition to patient-reported outcomes, multiple studies have examined the re-tear rates following MSC augmentation. One study found significantly lower re-tear rates (18% vs. 57%; *p* < 0.001) in the augmented repair group on one-year MRI scans [45]. Another study by Hernigou et al. found significantly lower re-tear rates at 6 months (100% vs. 67%) and 10 years (87% vs. 44%) for MSC-augmented rotator cuff repair compared to traditional repair [24]. Additionally, similar findings have been reported when examining the outcomes of PRP-augmented rotator cuff repair to traditional repair. A recent systematic review found that, although there were no significant patient-reported outcome differences, the PRP-augmented group demonstrated significantly lower re-tear rates at follow-up [46]. Although there is a paucity of evidence, the current literature examining MSCs for partial thickness rotator tears suggests that there are both significant improvements in pain and function as well as decreased future ret-ear rates. Of note, no previous studies have examined culture-expanded MSCs.

### Limitations

The results of this study must be understood within the confines of its limitations. Although an attempt was made to decrease heterogeneity through a strict inclusion protocol, many of the patients had multiple co-occurring pathologies. Due to this, many patients underwent treatment for more than one structure with various injectates (i.e., MSCs with PL and PRP). Given this, it was impossible to assess whether improvement in outcome measures was solely due to the treatment of the rotator cuff versus treatment of the co-occurring pathologies. Also, whether the observed clinical outcomes were due to the injected MSCs, PRP, PL, or a combination of the injectates utilized is unknown. Nonetheless, the preliminary results of this study suggest that an intratendinous injection of MSCs in combination with PRP/PL is effective regardless of co-occurring pathologies. Additionally, the lack of standardization of the doses of MSCs and PRP continues to be a constraint severely limiting the reproducibility of and conclusions on optimal cell dosing. As PRP, PL, and MSCs are autologous products, the specific concentrations and formulations vary among patients, thus making it difficult to examine the overall efficacy or compare outcomes with other comparative studies. It should also be mentioned that the low patient response rate observed after two years may have had a significant impact on data representation and led to selection bias as well. As previously mentioned, every patient had a minimum of five contact attempts via various methods in an effort to collect the data at each time point. Cost was an additional limiting factor. Currently, very few insurance companies provide coverage for biologic treatments. As such, the high cost associated with their preparation and administration continue to be a barrier to utilization and may limit accessibility for many patients as well as hinder broader applications of regenerative therapies. Lastly, the number of participants in this study was small, and there were no control groups. Additionally, a large proportion of the participants were male (73.33%). Thus, this sample may not accurately reflect the broader population with partial-thickness rotator cuff tears.

## 5. Conclusions

The results of this study show both continued safety and efficacy of culture-expanded MSCs for the treatment of partial-thickness rotator cuff tears. Treatment with autologous MSCs, PRP, and PL effectively demonstrated improvement in functional and pain metrics with no serious adverse events occurring during the 4 year follow-up period. Although the results of this study are promising, the limitations of this study highlight the continued need for additional large-scale randomized controlled trials (RCTs). The outcomes of this dataset suggest the efficacy of regenerative medicine techniques for the treatment of partial-thickness rotator cuff tears and further provide the methodological foundation through which future RCTs can be developed. This study reinforces the importance of continued research into regenerative medicine approaches as potential treatment for partial-thickness rotator cuff tears in order to give patients increased access to safe and effective alternative treatment options.

## Figures and Tables

**Table 1:** Patient demographics, diagnosis, and intervention performed. Abbreviations: M; male, F; female, MSCs; mesenchymal stem cells, PRP; platelet-rich plasma, PL; platelet lysate, GH; glenohumeral joint, OA; osteoarthritis, AC; acromioclavicular, M; million.

Demographics			Diagnosis	Procedure		
Patient	Gender	Shoulder (L/R/Bil)		Injectate	Rotator Cuff MSC Dose (Millions)	Additional Structures Injected
1	M	L	Supraspinatus tendinosis w/chronic interstitial tearing, labral fissuring, Grade 3 GH OA	MSCs, PRP, PL	2.0	Glenohumeral joint 15M MSC, labrum 2M MSC
2	M	R	Supraspinatus and subscapularis partial tearing and tendinosis, circumferential labral tear, Grade 1 GH OA	MSCs, PL	4.0	Glenohumeral joint 10M MSC, labrum 2M MSC
3	M	R	Supraspinatus/infraspinatus/subscapularis tendinosis and partial tearing, bursitis, AC OA with impingement	MSCs, PL,	5.0	Glenohumeral joint and AC joint 10M
4	M	R	Severe supraspinatus and infraspinatus tendinosis with partial tearing, Grade 1 GH OA, Advanced AC OA	MSC, PRP, PL	15.0	Labrum and AC joint
5	M	Bil	Partial tearing of supraspinatus, supraspinatus/infraspinatus/subscapularis tendinosis	MSC, PRP, PL	5-R 4-L	None
6	M	L	Partial tearing of subscapularis, biceps tendinosis	MSC, PL, PRP	2.25	None
7	M	L	Partial tearing of supraspinatus with tendinosis	MSC, PL, PRP	1.0	None
8	F	Bil	Partial tearing of supraspinatus, supraspinatus/infraspinatus/subscapularis tendinosis, biceps tendinosis, GH OA	MSC, PL,	2.0-R 2.0-L	Glenohumeral joint 10M MSC
9	M	L	Supraspinatus and infraspinatus tendinosis with partial tearing, bicep tendinosis, GH and AC joint OA	MSC and PL	5.0	Glenohumeral joint and AC joint 8M
10	M	R	Partial tearing of supraspinatus and subscapularis, labral fissuring	MSC, PL	5.0	Labrum 5M
11	F	L	Supraspinatus/infraspinatus/subscapularis tendinosis and partial tearing, labral fissuring	MSC, PL, PRP	1.0	Labrum 1M
12	F	R	Partial tearing of supraspinatus, biceps tendinosis, GH OA	MSC, PL	6.0	None
13	M	L	Supraspinatus tendinosis with partial tearing	MSC, PL	1.5	None
14	M	L	Supraspinatus tendinosis with partial tearing	MSCs, PL	2.0	None
15	F	R	Supraspinatus/infraspinatus/subscapularis tendinosis and partial tearing, GH and AC joint OA	MSCs, PL, PRP	14.0	Glenohumeral joint and AC join 20M, labrum- 14M
16	M	R	Supraspinatus/infraspinatus/subscapularis tendinosis and partial tearing, Bicep tendinosis, GH OA	MSCs, PL, PRP	5.0	Glenohumeral joint 15M
17	M	Bil	Supraspinatus/infraspinatus/subscapularis tendinosis and partial tearing, Bicep tendinosis, GH OA	MSCs, PL	10.0-R 10.0-L	Glenohumeral joint 20M
18	F	L	Supraspinatus/infraspinatus/subscapularis tendinosis and partial tearing	MSCs, PL, PRP	3.0	None
19	M	Bil	Supraspinatus and subscapularis partial tearing and tendinosis, biceps tendinosis, Grade 1 GH OA, AC Joint OA	MSCs, PL, PRP	10.0-R 2.0-L	Right glenohumeral joint 10M MSC, labrum 5M MSC, AC joint 1M MSC, left glenohumeral joint 2M MSC, labrum 4M MSC, AC joint 5M MSC
20	M	L	Partial Tearing of Supraspinatus, AC Joint OA	MSCs, PL, PRP	2.0	None
21	F	L	Supraspinatus/infraspinatus/subscapularis tendinosis and partial tearing, Bicep tendinosis	MSCs, PL	5.0	Labrum 2M MSCs
22	F	R	Supraspinatus/infraspinatus/Teres Minor tendinosis and partial tearing, Labral Fissuring	MSCs, PL	4.0	Labrum 1M MSCs
23	M	L	Supraspinatus/infraspinatus/subscapularis tendinosis and partial tearing, GH OA	MSCs, PL	4.0	Glenohumeral joint 10M MSC, AC joint1M MSC
24	M	L	Supraspinatus and infraspinatus tendinosis with partial tearing, Biceps tendinosis	MSCs, PL	4.0	None
25	F	Bil	Supraspinatus and subscapularis partial tearing and tendinosis, biceps tendinosis	MSCs, PL	2.0-R 2.0-L	None
26	M	L	Supraspinatus and subscapularis partial tearing and tendinosis, AC Joint OA	MSCs, PL	2.0	AC joint
27	M	Bil	Supraspinatus/infraspinatus/Teres Minor tendinosis and partial tearing	MSCs, PL, PRP	5.0-R 3.0-L	None
28	M	R	Supraspinatus/infraspinatus/subscapularis tendinosis and partial tearing	MSCs, PL	4.0	None
29	M	Bil	Supraspinatus tendinosis with partial tearing, GH and AC joint OA, labral fissuring right, supraspinatus tendinosis with partial tearing left	MSCs, PL	2.0-R 2.0-L	Right glenohumeral joint 10M MSC, labrum 4M MSC, AC joint 1M MSC
30	M	L	Supraspinatus and subscapularis partial tearing and tendinosis, biceps tendinosis	MSCs, PL, PRP	2.0	None

**Table 2:** The frequency of injection to each of the 4 rotator cuff tendons as well as other shoulder structures.

Injection Site	Number of Shoulders (%)
Rotator Cuff Tendon	
Supraspinatus	35 (94.6)
Infraspinatus	15 (40.5)
Subscapularis	18 (48.6)
Teres Minor	3 (8.1)
Glenohumeral Joint	13 (35.1)
Labrum	10 (27.0)
Acromioclavicular Joint	8 (21.6)

**Table 3:** Each outcome at baseline, 1, 6, 12, and 18 months, and 2, 3, and 4 years. Wilcoxon signed-rank tests were used to assess whether post-treatment scores differed significantly from baseline. For SANE scores, the baseline was considered the 1-month scores. Significance was defined as a *p*-value less than 0.05. Bonferroni correction was applied within each outcome measure. Significant *p*-values are bolded. Cohens’ d effect sizes are provided. Abbreviations: NRS; Numeric Rating Score, DASH; Disabilities of the Arm, Shoulder, and Hand, SANE; Single Assessment Numeric Evaluation.

Outcome	Mean	SD	N	*p*-Value	Cohen’s d
NRS					
Baseline	3.00	2.22	37	NA	NA
1 Month	3.04	1.86	27	0.31	0.23
3 Months	1.86	1.28	21	**<0.01**	0.9
6 Months	2.48	2.06	21	**0.05**	0.46
12 Months	1.11	1.52	19	**<0.01**	1.06
18 Months	1.29	1.64	14	**<0.01**	0.99
24 Months	0.75	1.14	12	**0.01**	1.05
3 Years	0.50	0.84	6	0.06	1.43
4 Years	1.00	NA	1	NA	NA
DASH					
Baseline	26.48	15.48	35	NA	NA
1 Month	26.70	16.14	24	0.59	0.13
3 Months	17.81	12.41	19	**<0.01**	1.05
6 Months	16.31	16.42	21	**<0.01**	1.19
12 Months	8.72	12.02	15	**<0.01**	1.42
18 Months	10.26	12.02	13	**<0.01**	2.36
24 Months	6.00	6.95	10	**<0.01**	2.00
3 Years	7.08	9.90	6	**0.04**	2.65
4 Years	5.83	NA	1	NA	NA
SANE					
1 Month	17.32	34.49	28	NA	NA
3 Months	46.50	33.76	20	**<0.01**	1.23
6 Months	53.1	31.20	21	**<0.01**	1.09
12 Months	56.65	36.68	17	**<0.01**	0.96
18 Months	66.25	30.39	12	**<0.01**	1.14
24 Months	62.73	31.17	11	**<0.01**	1.37
3 Years	89.17	11.14	6	**0.04**	2.36
4 Years	75.00	NA	1	NA	NA
6 Years	92.86	11.13	7	**<0.01**	2.19

**Table 4:** Individual patient 6-year SANE outcomes. Abbreviations: L; left, R; right.

Patient (Laterality)	6-Year SANE (%)
9	100
13	90
23 (L)	100
23 (R)	70
25	100
28	100
29	90

## Data Availability

The raw data supporting the conclusions of this article will be made available by the authors on request.

## Abbreviations

The following abbreviations are used in this manuscript:

NRS	Numeric Rating Scale
DASH	Disabilities of the Arm, Shoulder and Hand
SANE	Single Assessment Numeric Evaluation
PRP	Platelet-Rich Plasma
PL	Platelet Lysate
MSC	Mesenchymal Stem Cell
